# Application of SPF moisturisers is inferior to sunscreens in coverage of facial and eyelid regions

**DOI:** 10.1371/journal.pone.0212548

**Published:** 2019-04-03

**Authors:** Elizabeth A. J. Lourenco, Liam Shaw, Harry Pratt, Georgia L. Duffy, Gabriela Czanner, Yalin Zheng, Kevin J. Hamill, Austin G. McCormick

**Affiliations:** 1 Department of Eye and Vision Science, Institute of Ageing and Chronic Disease, University of Liverpool, Liverpool, United Kingdom; 2 Department of Applied Mathematics, Faculty of Engineering and Technology, Liverpool John Moores University, Liverpool, United Kingdom; 3 Department of Ophthalmology, Aintree University Teaching Hospital, Liverpool, United Kingdom; University of Naples, ITALY

## Abstract

Many moisturisers contain sun protection factors (SPF) equivalent to those found in sunscreens. However, there is a lack of research into how SPF moisturiser application compares to sunscreens in terms of coverage achieved and protection afforded. Previously we demonstrated that users incompletely covered their eyelid regions during routine sunscreen application. Here, we aimed to determine if SPF moisturiser users also displayed these tendencies. A study population of 84 participants (22 males, 62 females, age 18–57) were exposed to UV radiation and photographed using a tripod mounted UV sensitive DSLR camera on two separate visits. At visit one, images were acquired before and after applying either SPF30 sunscreen or moisturiser, then at visit two the study was repeated with the other formulation. Images were processed for facial landmark identification followed by segmentation mapping of hue saturation values to identify areas of the face that were/were not covered. Analyses revealed that application of moisturiser was significantly worse than sunscreen in terms area of the whole face missed (11.1% missed with sunscreen compared to 16.6% for SPF moisturiser p<0.001 paired t-test). This difference was primarily due to decreased coverage of the eyelid regions (14.0% missed with sunscreen, 20.9% moisturiser, p<0.001). Analysis of a post-study questionnaire revealed participants to be unaware of their incomplete coverage. Secondary analyses revealed improved coverage in males (p = 0.05), and, with moisturiser only, in participants with darker skin tones (p = 0.02). Together these data indicate that, despite potential advantages in terms of increased frequency of application of moisturiser, the areas of the face that are at higher cancer risk are likely not being protected, and that participants are unaware that they are at risk. As such, alternative sun-protection strategies should be promoted.

## Introduction

Use of sun protection factor (SPF) containing products is widely promoted to protect against the harmful effects of ultraviolet radiation exposure [1,2,3]. In spite of many public health initiatives, incidences of both melanoma and non-melanoma skin cancer are increasing [4]. Traditionally, manufacturers have focused on delivering speciality SPF formulations marketed as sunscreens. However, there has more recently been an increased availability of alternative SPF formulations, most notably in daily moisturisers. Although initially the SPF rating of these formulations was lower, many widespread brands now market their product with SPF in the 30 to 50 range, equivalent to the level promoted for traditional sunscreens. While these products will almost certainly lead to increased sun-protection in those that do not regularly use sunscreens, it has yet to be formally evaluated whether the manner in which SPF moisturisers are applied will provide sufficient protection to replace traditional sunscreens. This information is important as behavioural changes may occur in response to a perception of being protected.

Recently, we modified a DSLR camera to only respond to UV radiation and then used this camera to assess routine sunscreen application in a group of university students. These studies revealed that participants were disproportionately poor at applying sunscreen to the regions around the eyelids compared with the rest of their face [5]. These findings are of particular importance as not only is there a disproportionally high incidence of melanoma and non-melanoma skin cancers occurring on the head and neck compared with the rest of the body, but also as the eyelid skin displays the highest skin cancer incidence per unit area [6,7]. Moreover, a recent report has indicated that the incidence of squamous cell carcinoma of the eyelids is also still increasing [4]. Of particular importance is the medial canthal region, the area between the medial end of the eyelids and the nose, as this region is at high risk area for basal cell carcinoma, both in terms of the frequency and the severity of the disease [8,9,10,11]. Our previous research has shown the medial canthal area to be the least effectively covered region of the face [5]. One reason cited by participants for failing to cover their eyelids effectively was concern over stinging associated with getting sunscreen in the eyes. We hypothesised that this fear would be less in SPF moisturiser formulations, and that this in turn would result in improved, more comprehensive, coverage.

In this study we have compared the application effectiveness in terms of area of the face covered or missed when using SPF moisturisers compared with traditional sunscreen.

## Materials and methods

### Ethics

**Fig 1:**
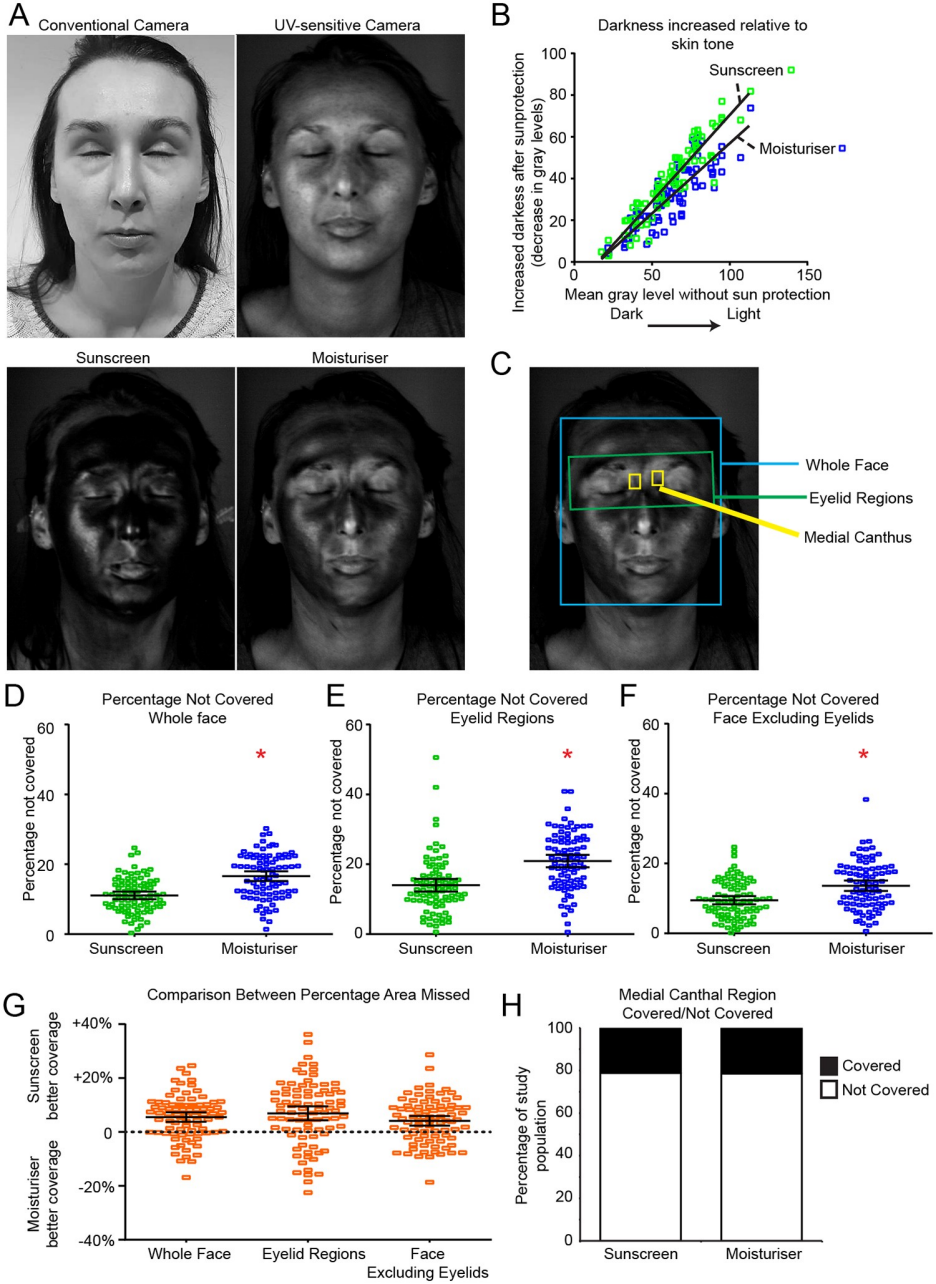
Increased areas of the face and eyelid regions are missed using SPF moisturiser compared with sunscreen. A. Representative images from the same person imaged using a conventional camera (top left), UV-sensitive camera (top right), UV-sensitive camera after application of SPF30 sunscreen (bottom left) and UV-sensitive camera after SPF30 moisturiser application (bottom right). B. Scatter graph of increase in image darkness relative to skin tone. C. Image segmentation for application analysis, boxes represent areas analysed; blue = face, green = eyelid, yellow = medial canthus. D, E and F dot plots of percentage missed with sunscreen and moisturiser in indicated areas. Black lines = mean and SD, n = 84. * indicates significant difference P<0.05 between groups, determined by paired t-test (D) or repeated-measures ANOVA (E and F). G. Within individual difference in area missed between moisturiser and sunscreen, positive scores = more missed after moisturiser application. H. Stacked column graph displaying medial canthus coverage scored as fully covered/missed, plotted as percentage of population.

Ethics were obtained from The University of Liverpool Ethics Review board, reference number 201606181. Participants gave written informed consent for the study. The individuals pictured in Fig 1 and S4 Fig have provided written informed consent (as outlined in PLOS consent form) to publish their image alongside the manuscript.

### Subject recruitment

84 participants were recruited, predominantly from the University of Liverpool, via interdepartmental emails to students, and display of recruitment posters across campus. Participants were excluded if they had previously participated in, or were aware of any other UV-imaging study, and if they had any allergies or sensitivities to either sunscreens or SPF moisturisers. There were no additional exclusion criteria for ethnicity, occupation, or any other demographics. Financial remuneration was offered upon study completion along with the option to receive a copy of any images.

### Study design

This was a prospective observational study comparing application efficacy between sunscreen and SPF moisturiser. Test sunscreen and SPF moisturiser were selected based on reported market share in the UK [12,13]. Products tested had the same advertised SPF and the same primary active ingredient, titanium dioxide; Olay Regenerist 3 Point Moisturiser SPF30 (hereafter, “SPF moisturiser”, Procter & Gamble Co, Cincinnati, Ohio, US), and Soltan sensitive hypoallergenic suncare lotion SPF30 (hereafter “sunscreen” Boots UK Ltd, Nottingham, UK). It should be noted that, in addition to titanium dioxide, these formulations each contain additional UV absorbing chemicals (SPF moisturiser; Ethylhexyl Methoxycinnamate, Butyl Methoxydibenzoylmethane, and Ethylhexyl Methoxycrylene, sunscreen: Octocrylene, Butyl methoxydibenzoylmethane, Bis-ethylhexyloxyphenol methoxyphenyl triazine, Ethylhexyl salicylate, and Diethylhexyl butamido triazone). Both products were suitable for application to the face and did not specify for the users to avoid the eyelids, though both recommend avoiding the eyes.

Participants attended on two separate dates. During session 1 they completed a pre-study questionnaire (S1 Fig) and were imaged after applying either sunscreen (N = 60) or SPF moisturiser (N = 24). At session 2 subjects were imaged after applying the other formulation, they then completed a post-study questionnaire; answering part A before seeing images, and part B after (S2 Fig). At each visit, participants were initially imaged to determine if they were already wearing any SPF containing products. Where necessary, participants were asked to remove that product and then reimaged to provide a baseline reading prior to sunscreen or SPF moisturiser application. At both study visits, participants received the following instruction; “please apply this as you normally would”. A mirror was available for use at the participants’ discretion. On the second visit, after image acquisition and completion of part A of the post-study questionnaire had occurred, the participant and researcher discussed the photos focusing on areas missed and thickness of application. They were then taught ways that they could improve their technique, and how to better look after their skin to reduce risk of premature ageing and skin cancer. Following the discussion participants filled out part B of the questionnaire.

### Imaging

Participants were exposed to a UV-A radiation from a 2x LED emitting UV stand (OPPSK LED-BAR 9X3W, 110-240W 51x7x7cm) and photographed used a tripod mounted DSLR camera (Canon EOS Rebel XTi 400D) with a 60mm EF-S macro lens (both Canon, Surrey, UK) using settings F3.5, shutter speed 0.8s. This camera was modified to record only UV radiation through removing and replacing the internal hot mirror with a UV band-pass filter (Lifepixel, Mukilteo, WA, USA).

To determine if the sunscreen and SPF moisturiser used lead to equivalent increased darkness of image in our camera/lighting set up, a dose-response curve was generated through 4 participants Appling equal mass (50 mg, 100 mg, 250 mg per half of face) of sunscreen and moisturiser to half of their face (2x sunscreen on left-hand side, moisturiser right, 2x sunscreen right, moisturiser left). The mean pixel intensities were determined using FIJI (NIH, Bethesda, Maryland, USA) by selecting the facial region stretching from the top of the forehead to tip of the chin, and extending to the outer boundaries of the eyes, then dividing this region in half vertically.

To calculate percentage coverage, images were analysed using an automated system to identify areas of the skin that had not been covered by the product using a previously described method [5,14]. Briefly, facial landmarks were detected using the dlib package (http://dlib.net); these landmarks were used to define the face and eyelid regions, and to classify the medial canthus. Binary segmented versions of the images were created by mapping to hue saturation values. From the segmented images, pixels were assigned a value depending on whether they were covered by the product. This was converted to a percentage of pixels uncovered within the predetermined area of the participants face to give an overall percentage of area missed.

### Rheology

Rheological measurements were performed using an Anton Paar MCR 302 Rheometer (Anton Paar, Hertford Herts, UK) with a cone-plate configuration of diameter = 6 cm and 1° cone angle. The cone height was set to 60.0 mm while 1–2 mL of each sample was loaded onto the plate, then lowered to 0.118 mm and the samples trimmed to remove excess. Measurements were acquired over a shear rate logarithmically increasing scale between 0.01 and 1000 s^-1^. All measurements were taken at a constant 37 °C to mimic substance behaviours at body temperature. Rheocompass software was used to collect the data using a viscoelastic liquids flow curve program. For each substance, measurements were performed in triplicate.

### Data analysis

Data were analysed using SPSS (IBM Corp. Released 2013. IBM SPSS Statistics for Windows, Version 22.0. Armonk, NY: IBM Corp). The primary study question was to determine if there were differences between SPF formulations in terms of area missed. Normality was tested using Kolmogorov-Smirnov test. If normally distributed, data were assessed using a paired t-test or repeated measures ANOVA. If data were not normally distributed, and normality could not be achieved through log or square root transformation, then a non-parametric Mann-Whitney U test was used. To control for order effects, the sunscreen first and moisturiser first groups were independently analysed (S3 Fig). Effects were consistent between groups therefore data were pooled for all subsequent analyses. Skin type was evaluated on four levels I, II, III and IV, then, due to low numbers of darker skin tones, collapsed into two categories for analysis; type I and II vs III and IV. We considered the confounding characteristics of sex, skin-type and age in applying the sunscreen and SPF moisturiser. To control for the possible confounding effect of sex and skin type we performed independent t-tests in subgroup analyses. Furthermore, for sex, age and skin type a univariate ANCOVA with two-factors and covariate was used to test for the main effect of factors and for the interaction. We found the main effect of age and the interactions not-significant, hence they were not used in the final analyses. All tests were performed at a significance level of 0.05.

## Results

In order to compare sunscreen and SPF moisturiser application habits we recruited 84 participants (62 female, 22 male, aged 18–57 years) to a two-visit trial. At each visit the participants were instructed to apply either sunscreen or moisturiser in their normal manner: without direction in terms of mode of application or volume to apply. They were then imaged under UV-A radiation with a UV-sensitive camera (Fig 1A). Casual observation of the acquired images suggested that the participants appeared darker after sunscreen application than moisturiser. Differences in terms of chemical formulation between the sunscreen and SPF moisturiser raised the possibility that they would perform differently in terms of absorption of the UV-A radiation used in our imaging approach. We therefore generated a dose-response curve by applying a range of known quantities of moisturiser or sunscreen side-by-side to each half of participants’ faces and measuring the mean pixel intensity of each half (S4 Fig). These analyses revealed the moisturiser used has slightly superior absorption properties per unit mass when imaged using our lighting and camera set-up; however, these differences were not statistically significant (p>0.05). With this caveat to interpretation in mind, we performed analysis of the change in mean pixel intensity in our participants (Fig 1B). These data confirmed that the increased light absorption in the sunscreen images was greater than in those taken after SPF moisturiser application (linear regression, F 7.47, P<0.01), suggesting that overall less product was applied during SPF moisturiser application. Note that we cannot directly infer relative levels of UV-B protection from these data; only that less SPF moisturiser appears to have been applied.

Next, the acquired images were segmented based on facial landmarks to identify the face, eyelid and medial canthal areas (Fig 1C). The images were then analysed using a previously described algorithm to identify the percentage of these areas that were covered/not covered [5]. Comparison between sunscreen and SPF moisturiser images revealed the same participants performed worse, missing greater areas, when they applied SPF moisturiser (Fig 1D to 1G mean 16.6% SD 6.4 missed when using moisturiser compared with 11.1% SD 5.0 missed with sunscreen, paired t-test p<0.001). Specific sub-analysis of eyelid and non-eyelid regions revealed that the regions around the eyelids were particularly ineffectively covered with both sun-protection formulations. Again the moisturiser application lead to inferior coverage than the sunscreen (eyelid region; SPF moisturiser 20.9% SD 8.2, sunscreen 14.0% SD 8.3 Fig 1E and 1G, repeated measures ANOVA, p<0.001). Differences between formulations were smaller in the non-eyelid regions but SPF moisturise application still led to greater areas missed (moisturiser 13.6% SD 6.9, sunscreen 9.5% SD 5.5, P<0.01, Fig 1F and 1G). As basal cell carcinomas in the medial canthus are particularly prevalent and are associated with worse outcomes [8,9,10,11], we also specifically analysed these regions using a binary covered/non-covered scale. Both formulations were applied equally poorly; 66 of 84 (78%) of participants failed to cover the medial canthus (1H) and only 5 of the study group successfully covered this region in both visits. These findings are consistent with our previous report and again highlight this region as particular at-risk area of the face.

Subgroup analysis within the population revealed that males generally missed lower percentages than females, with differences reaching statistical significance between genders in the percentage missed in eyelid regions (moisturiser; male mean 16.7% SD 7.4, female 22.4% SD 8.0 P<0.01, sunscreen; male 10.7% SD 5.6, female 15.3% SD 8.8 P<0.05 Sidak’s multiple comparison test, S5 Fig). Those with darker skin tones generally achieved greater coverage than skin types I and II, with differences only reaching statistical significance with moisturiser in the eyelid regions (Type I and II mean 22.1% SD 8.5, Type III and IV mean 11.7% SD 5.8, P<0.05 S5 Fig).

Next, we performed rheology to determine if differences in viscosity between the formulations could be contributing to the differences in coverage achieved (S6 Fig). Both the SPF moisturiser and the sunscreen displayed profiles of shear thinning liquids; viscosity decreases with increasing shear rate. Importantly, this analysis revealed that the SPF moisturiser had a consistently higher viscosity than the sunscreen, indicating that it is harder to spread than the sunscreen (S6 Fig).

**Fig 2:**
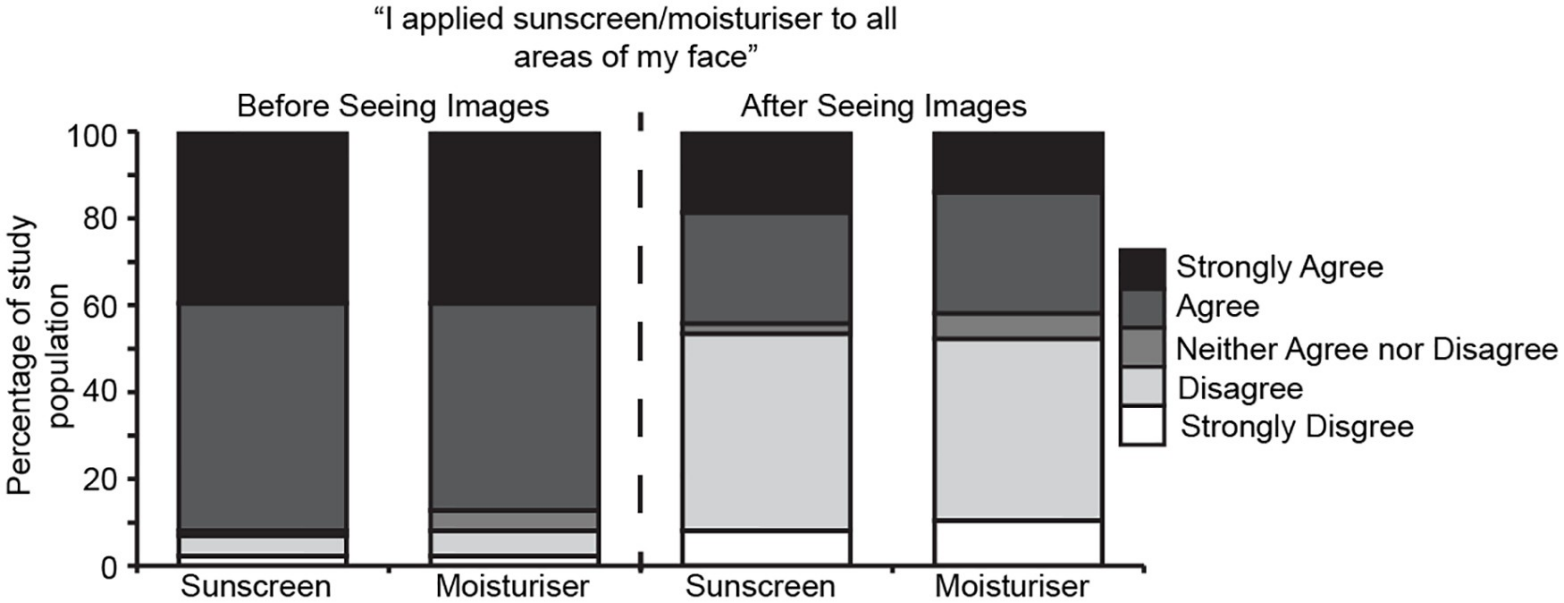
Participants were unaware of their failure to apply sunscreen or SPF moisturiser effective. Stacked column graph depicting questionnaire responses to the question “I applied sunscreen/moisturiser to all areas of my face”, before (left two columns) or after (right two columns) seeing the acquired images.

We were interested to determine if the participants were aware of the inferior coverage achieved using moisturiser. In a post-study questionnaire (S1 Fig) conducted prior to the participants seeing their images, we asked them to answer; “I applied (sunscreen or moisturiser) to all areas of my face”. The vast majority of participants responded to this question with “agree” or “strongly agree” with very little difference between their perceived application of sunscreen compared with moisturiser (77/84 sunscreen, 73/84 moisturiser responded “agree” or “strongly agree”, Fig 2, left columns). We next showed the participants their own images and asked them to answer the same question, rating their application of both compounds (Fig 2, right columns). In this self-assessment of application performance, more than half of the participants answered the question “disagree” or “strong disagree” (46/84 sunscreen, 45/84 moisturiser). These data indicate that incomplete coverage achieved was unlikely to be due to a conscious decision.

Further questionnaire responses indicated that participants underestimated how much non-visible sun-damage was present on their face (Table 1), and, after seeing the images, they responded that they intended to use sun protection more frequently in the future, will pay increased attention to frequently missed areas including the eyelids, and intend to wear sunglasses (Tables 1 and 2).

**Table 1:** Questionnaire responses.

**Skin Damage**	**Extremely Below Average**	**Below Average**	**Average**	**Above average**	**Extremely Above average**	
How damaged do expect your skin to be?	2	11	43	25	3	
	**Much Less**	**A bit less**	**The same**	**A bit more**	**Much More**	
Was your sun damage what you expected?	1	5	18	28	26	
**Sunscreen use**	**Every Day**	**Weekly**	**Monthly**	**Several times a year**	**Yearly**	**Never**
How regularly do your think about sun damage?	5	22	12	23	15	6
How often do you use sunscreen	4	6	9	49	9	7
After seeing these images how often will you use sunscreen	16	9	14	15	5	1

**Table 2:** Questionnaire responses.

	**Yes**	**No**	
Did you pay specific attention to your eye area?	25	59	
Do you currently use a SPF containing moisturiser?	18	66	
	**Yes**	**No**	**N/A**
Were you surprised at the areas you missed using sunscreen?	49	27	8
Were you surprised at the areas you missed using moisturiser?	52	27	4
In the future will you pay more attention to the areas you missed using sunscreen?	77	4	3
In the future will you pay more attention to the areas you missed using moisturiser?	74	6	3
	**Yes**	**No**	**Sometimes/Not sure**
After seeing these images will you use an SPF containing moisturiser?	54	5	25
Will you wear sunglasses to protect your eye area?	76	4	6

## Discussion

Together the data presented here demonstrates that areas of the face that are more vulnerable to skin cancer are also more likely to be missed during application of SPF moisturisers that with sunscreens, and, importantly, that people applying these products are unaware that they are failing to cover these at-risk regions.

An important point to emphasise is that the problems we are identifying here are a trend toward repeatedly missing the same areas, as opposed to a more general problem of incomplete coverage. It is important to emphasise that although there are widespread benefits to sun exposure ranging from activating vitamin D, and even improving moods in patients with seasonal affective disorder [15,16], it is the dangers of repeated UV-mediated DNA damage in the same region that thorough application of sun protection compounds protects against. Moreover, whereas short term UV exposure has health benefits and indeed are recommended by NICE [17,18,19], people may be inclined to spend more time in the sun when wearing SPF containing products and therefore, unprotected areas therefore are likely to receive increased cumulative UV doses.

Questions remain as to why the relatively inferior coverage was achieved by the same participants when they used moisturiser compared with when they applied sunscreen. The most likely explanation for the decreased overall protection lies simply with the volume of the substance applied. However, this does not necessarily explain the decreased area covered; the differences in the rheology or consistency of the products are likely a major contributor [20]. Indeed differences in the ability to spread the product will not only affect how well the product is applied but also the amount used. In addition to these physical properties, the product presentation in terms of packaging and labelling could also be relevant. In designing our study, our goal was to assess application behaviours in as close to real-world situations as possible. We deliberately refrained from providing any direction regarding volumes to apply and provided local (UK) market leading brands for the participants. However, this raises a potential confounder as moisturisers are generally sold in smaller volume containers, which may have an effect on the volume used per application.

An important point that we observed in our previous sunscreen studies and again in these current studies, is that participants were unaware of their relative inability to achieve complete coverage [5]. This suggests that the behaviour driving these observations is not a deliberate attempt to avoid the eyelid regions, but rather an unconscious behavioural difference. This finding is particularly important as it emphasises the need for public education in this area. This public health message must weigh the benefits of UV exposure against the risks of UV damage, and should focus the message on protecting the vulnerable eyelid and medial canthal areas.

## Conclusions

The addition of SPF to daily moisturisers has lots of potential advantages in terms of likely increase in general protection in all weather conditions. However, our data show that those potential advantages may be offset by incomplete coverage to areas at high risk of skin cancer and a mistaken belief that the face is fully protected. In many environments, the risk that you are unaware of poses the greatest danger and as such a more extensive public awareness campaign is warranted.

## Supporting information

S1 Fig Pre-study questionnaire. (DOCX)

S2 Fig Post-study questionnaire. (DOCX)

S3 Fig Order effect determination. Dot plot of percentage of indicated region missed in participants applying sunscreen at visit 1(sunscreen 1^st^ visit) and SPF moisturiser at visit 2 (Moisturiser 2^nd^ visit) or SPF moisturiser at visit 1 (moisturiser 1^st^ visit) and sunscreen at visit 2 (sunscreen 2^nd^). Black lines are mean and standard deviation.
(TIF)

S4 Fig Comparison of increased pixel intensity induced application of equal mass of sunscreen or SPF moisturiser. A. Representative images of participant after application of 0, 50 mg, 100 mg or 250 mg of sunscreen (left half of image) or SPF moisturiser (right half). B. Dot plot of mean grey level of pixels measured in the entire facial region after application of sunscreen or SPF moisturiser. N = 4.
(TIF)

S5 Fig SPF moisturiser vs sunscreen application comparison between gender (A) and skin tone (B). Dot plots of percentage of indicated areas missed using after sunscreen or SPF moisturiser application. Black lines are mean and standard deviation. * denote differences between groups are statistically significant as assessed by 1-way ANOVA and Sidak’s multiple comparison test.
(TIF)

S6 Fig Viscosity as a function of shear rate of SPF moisturiser and sunscreen measured at 37°C. Each dot represents one measurement at each shear rate, solid line indicating mean from three repeats.
(TIF)

## Abbreviations

BCC: Basal cell carcinoma
SCC: Squamous cell carcinoma
SPF: Sun protection factor
UV: Ultraviolet
