# Supplementary material for: Application of SPF moisturisers is inferior to sunscreens in coverage of facial and eyelid regions
Source: PLoS One. 2019 Apr 3;14(4):e0212548. doi: 10.1371/journal.pone.0212548 (PMC6447356; doi:10.1371/journal.pone.0212548)
Supplement: S1 Fig — (DOCX) [file pone.0212548.s001.docx]

**Pre study questionnaire**

**Name:**

**Date:**

Please circle answers

1. Sex

Male Female Prefer not to say

1. Age

……………………………………….

1. Occupation

………………………………………..

1. Skin type according to Fitzpatrick Scale

I II III IV V VI
