# Supplementary material for: Application of SPF moisturisers is inferior to sunscreens in coverage of facial and eyelid regions
Source: PLoS One. 2019 Apr 3;14(4):e0212548. doi: 10.1371/journal.pone.0212548 (PMC6447356; doi:10.1371/journal.pone.0212548)
Supplement: S2 Fig — (DOCX) [file pone.0212548.s002.docx]

**Post study questionnaire- Before seeing images**

**Name:**

**Date:**

**Please circle the statement that describes your opinion**

1. I applied the sunscreen to all areas of my face


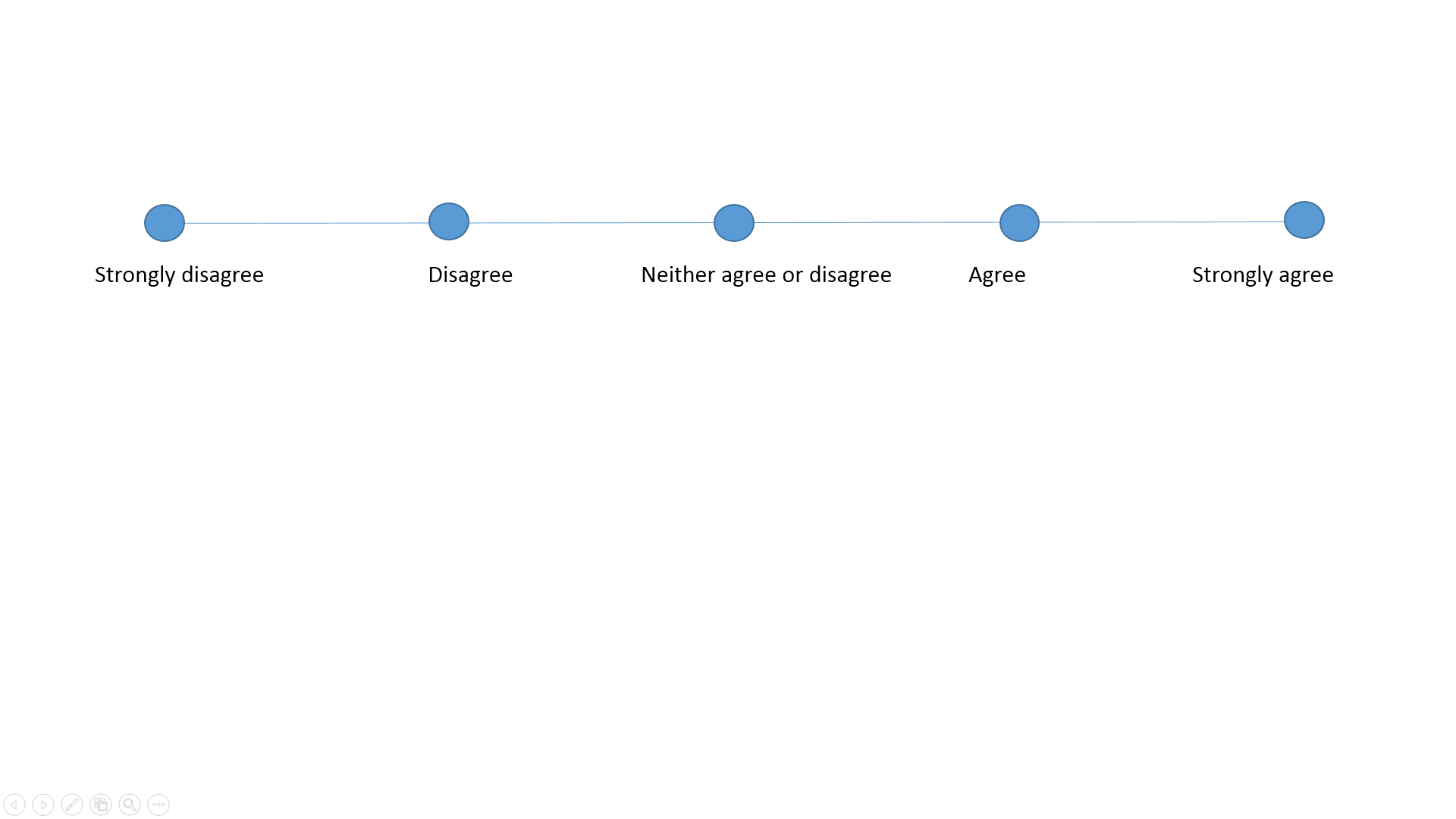


1. I applied the spf moisturiser to all areas of my face


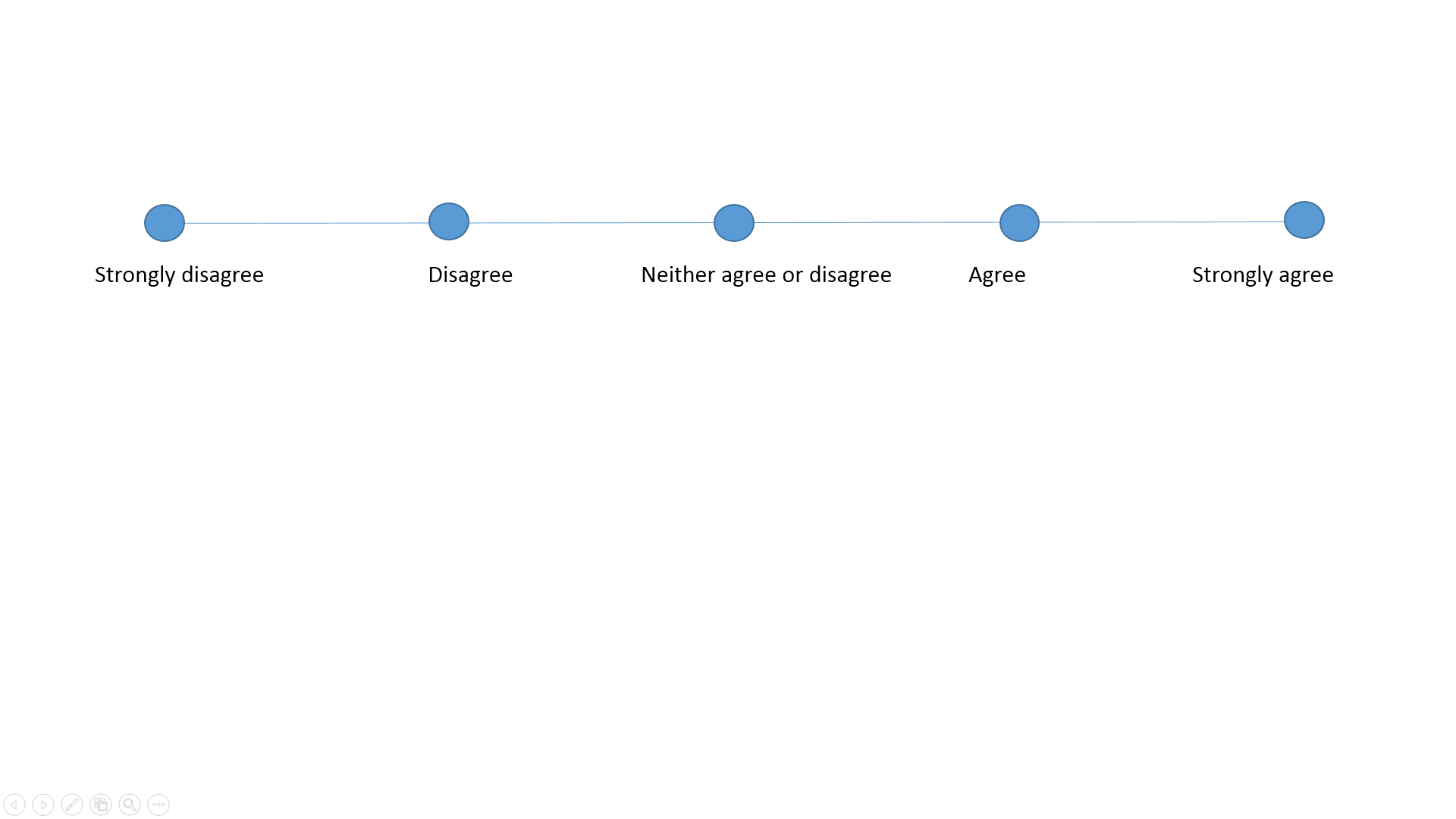


1. Did you pay specific attention to your eye area?

Yes No

1. How regularly do think about sun damage?


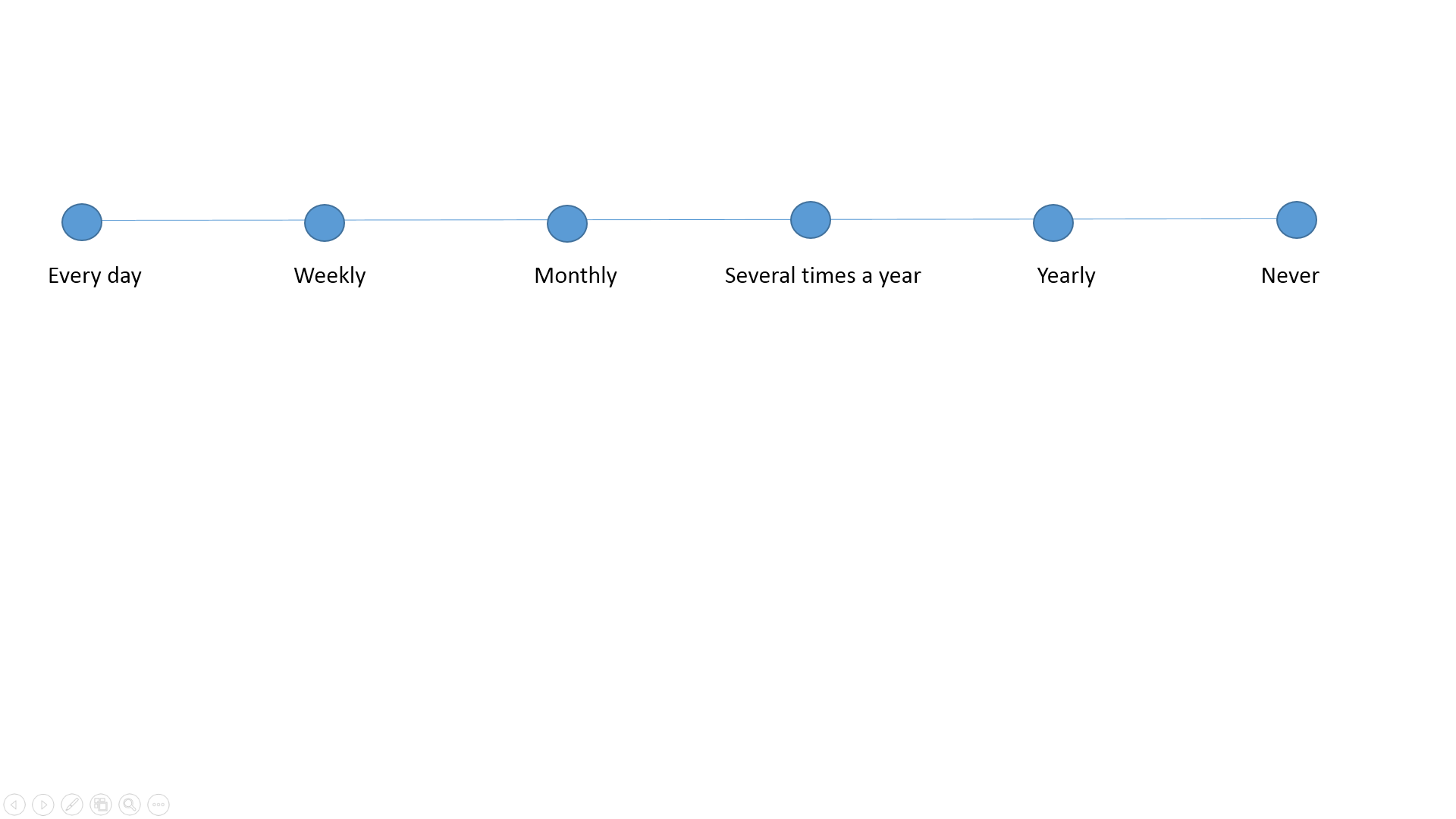


1. How damaged do you expect your skin to be?


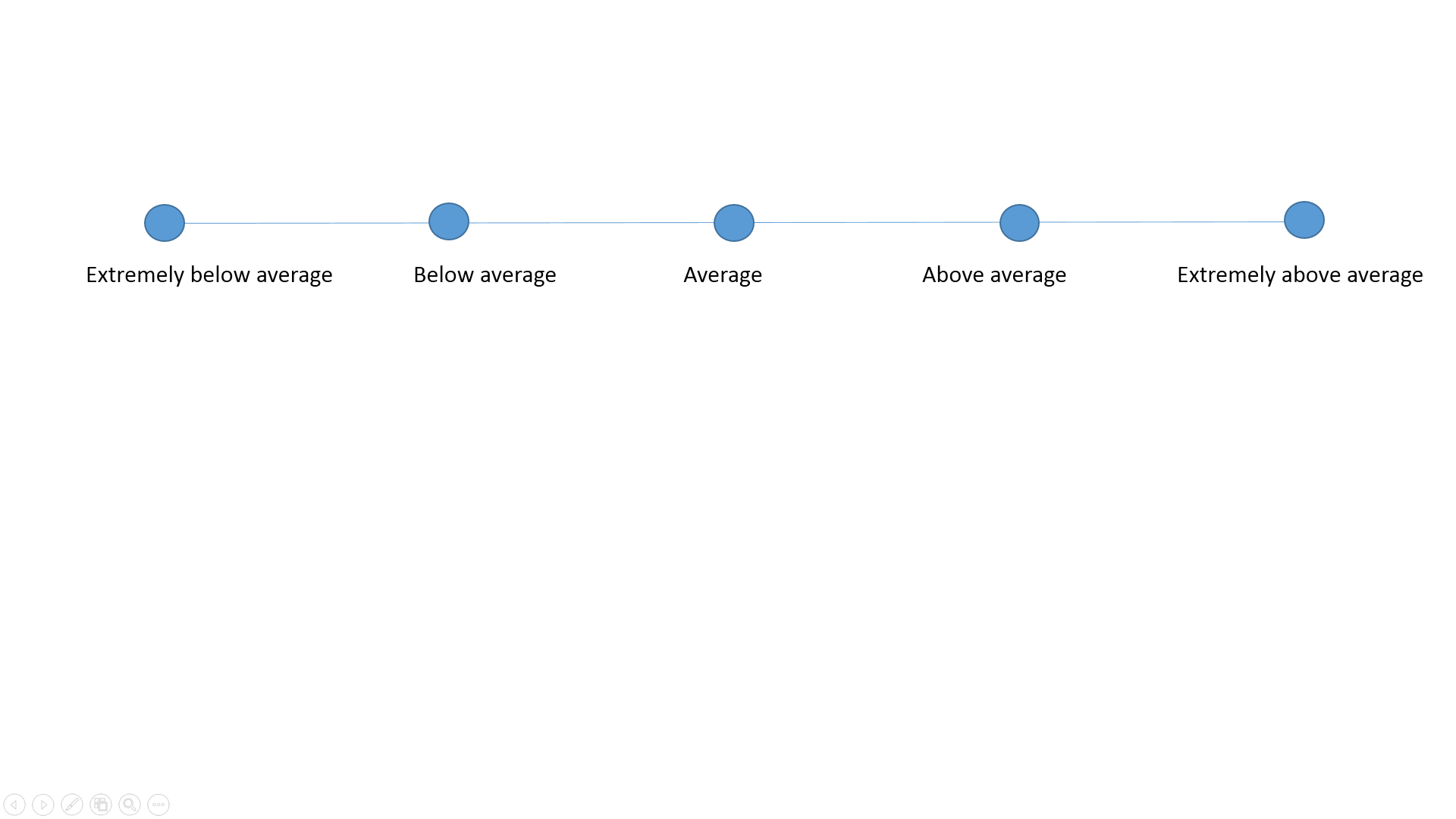


1. How often do you use sunscreen?


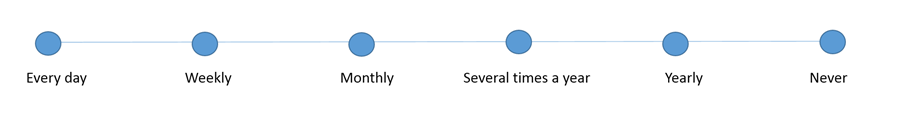


1. Do you currently use an SPF containing moisturiser?

Yes No

**Post study- After seeing images**

**Name:**

**Date:**

**Please circle the statement that describes your opinion**

1. I applied the sunscreen to all areas of my face


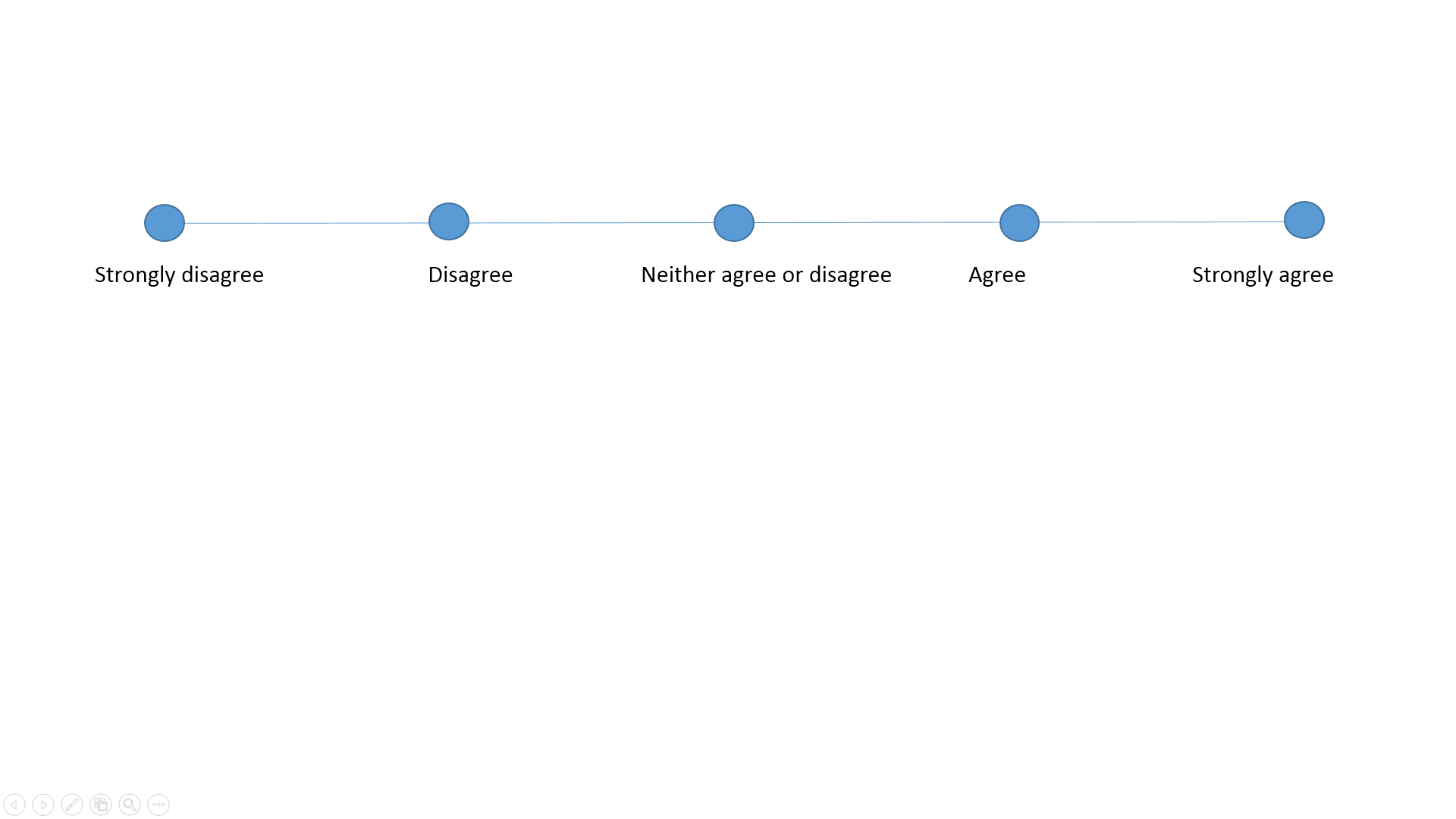


1. I applied the spf moisturiser to all areas of my face


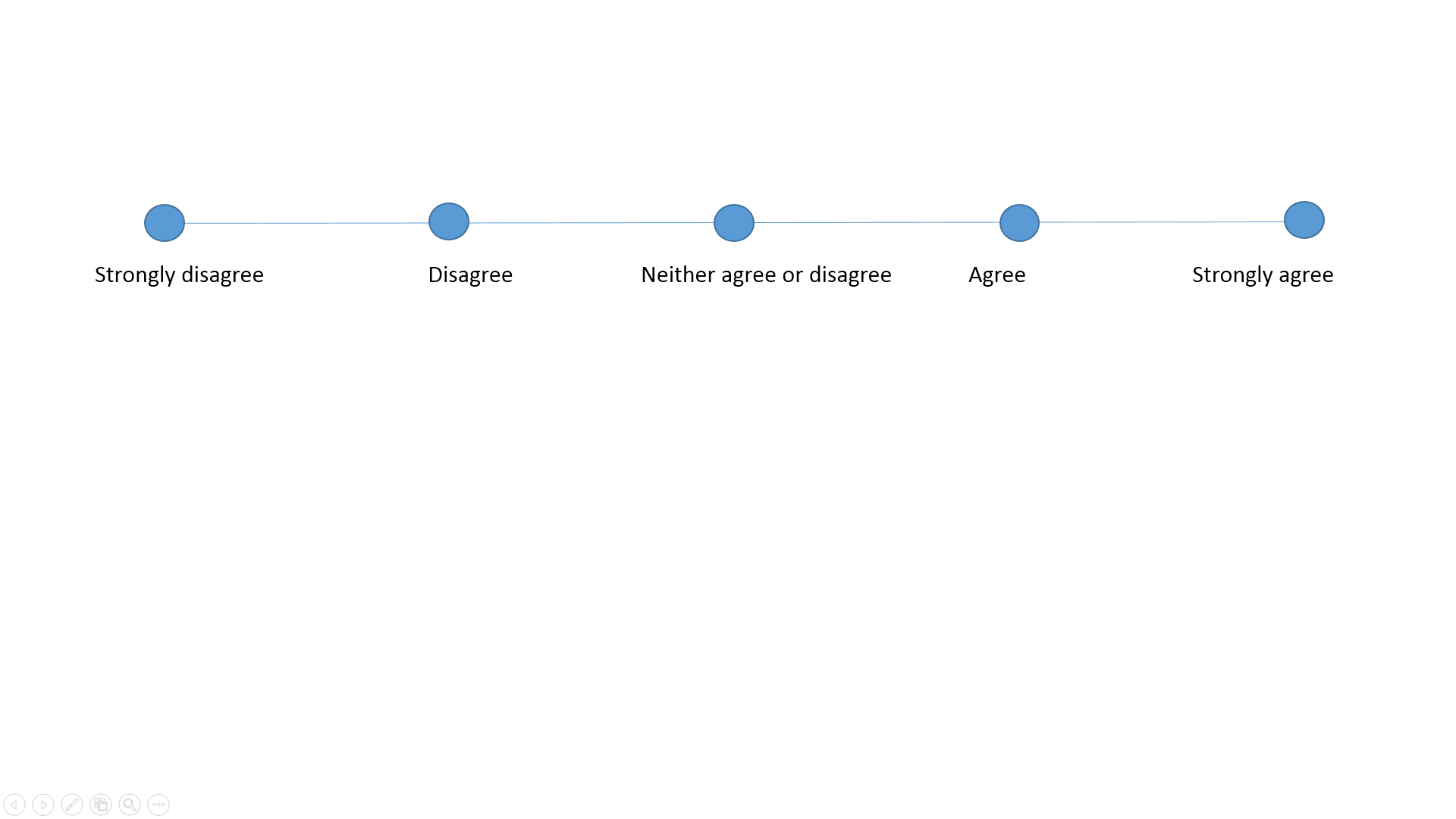


1. Were you surprised at the areas that you missed?
2. Sunscreen Yes No N/A
3. Spf moisturiser Yes No N/A
4. In the future will you pay more attention to the areas you missed?

a) Sunscreen Yes No N/A

b) Spf moisturiser Yes No N/A

5) Was your sun damage what you expected?
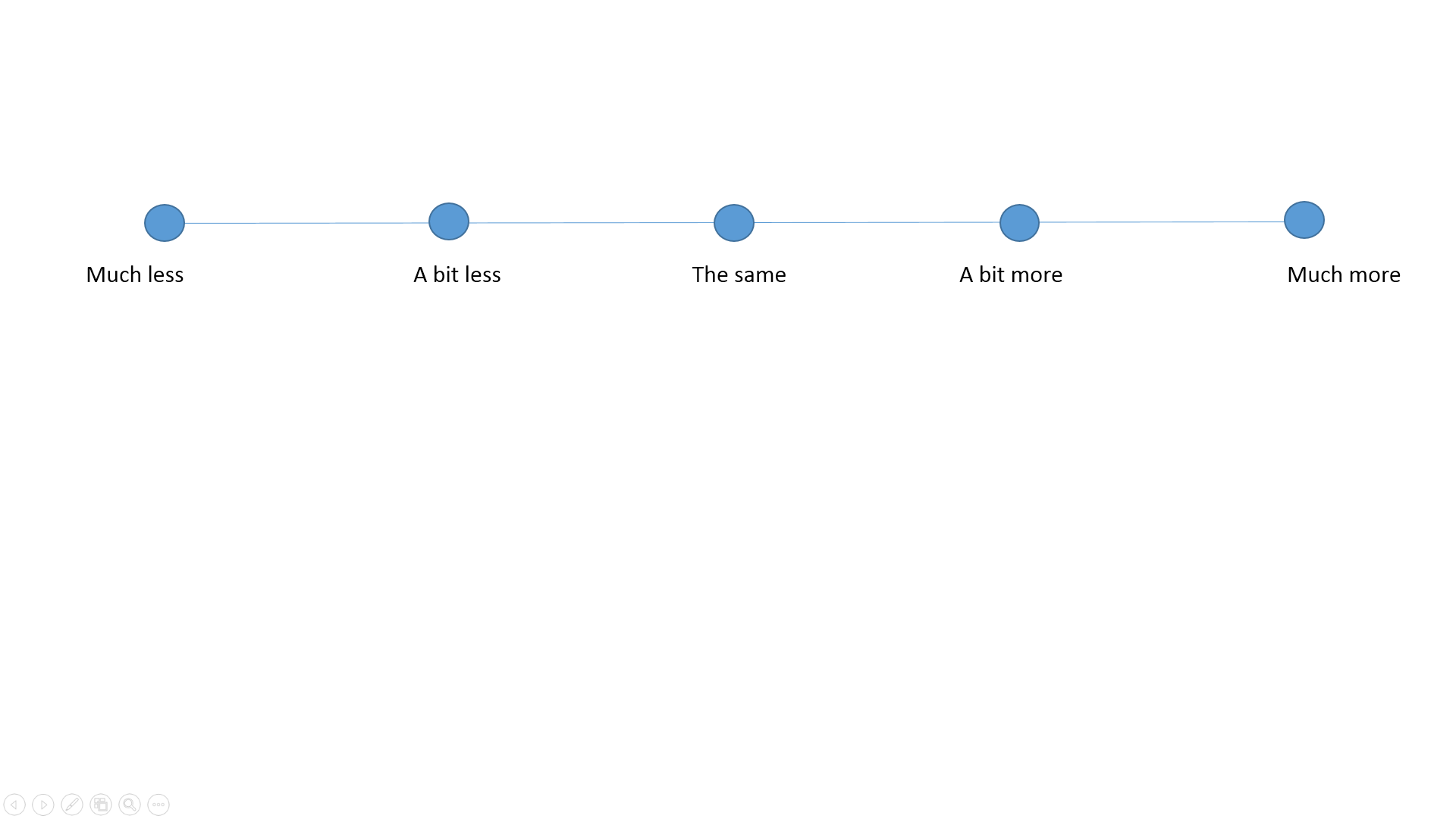


6) After seeing the images how often will you use sunscreen?


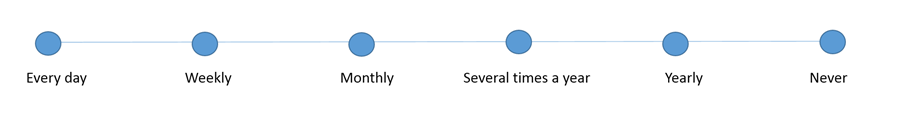


1. After seeing these images will you use an SPF containing moisturiser?

Yes No Sometimes Not sure

1. Will you wear sunglasses to protect your eye area?

Yes No Not sure
