# Supplementary figures and images for: Application of SPF moisturisers is inferior to sunscreens in coverage of facial and eyelid regions
Source: PLoS One. 2019 Apr 3;14(4):e0212548. doi: 10.1371/journal.pone.0212548 (PMC6447356; doi:10.1371/journal.pone.0212548)

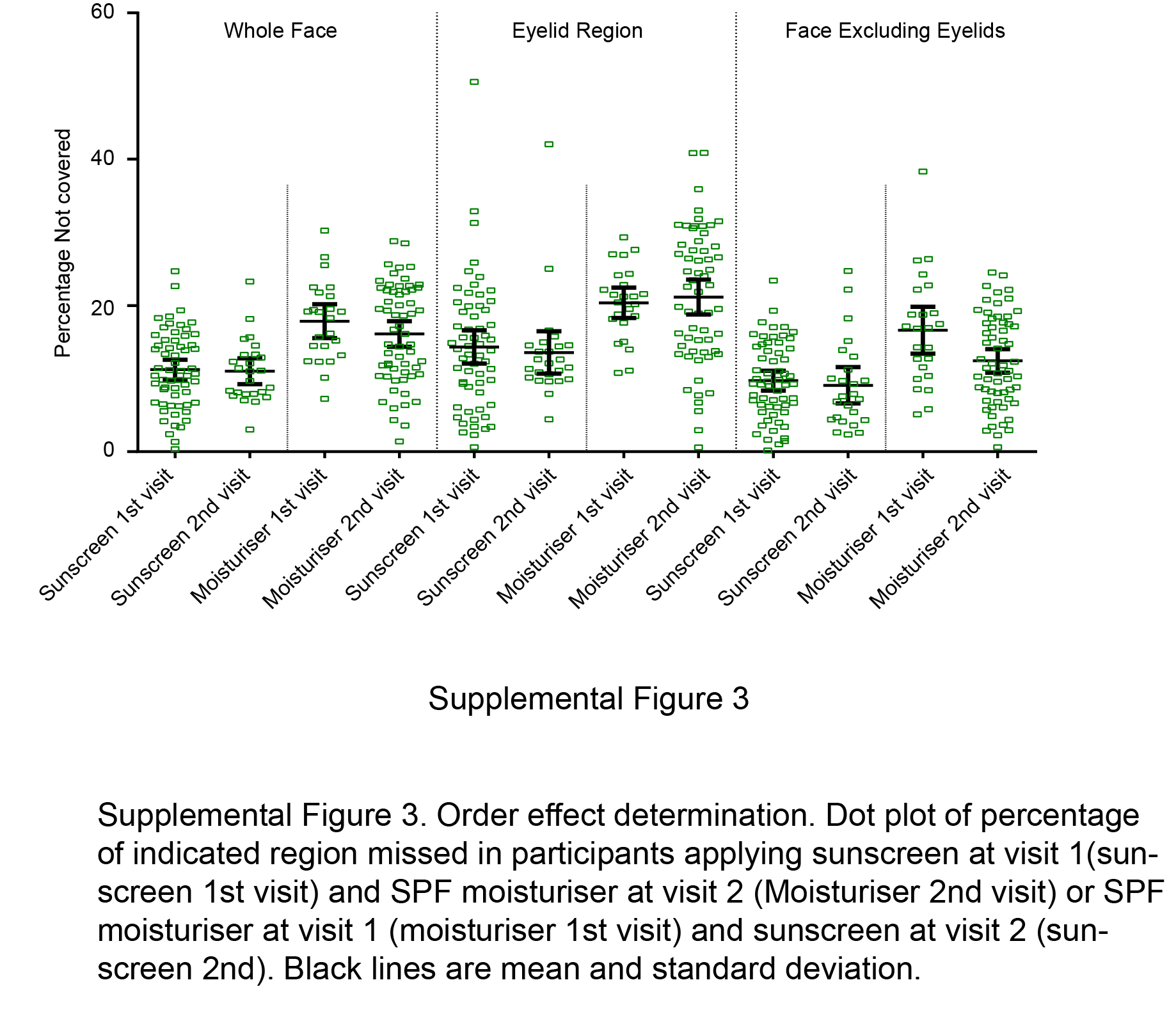

Supplement: S3 Fig — Dot plot of percentage of indicated region missed in participants applying sunscreen at visit 1(sunscreen 1st visit) and SPF moisturiser at visit 2 (Moisturiser 2nd visit) or SPF moisturiser at visit 1 (moisturiser 1st visit) and sunscreen at visit 2 (sunscreen 2nd). Black lines are mean and standard deviation. (TIF) [file pone.0212548.s003.tif]

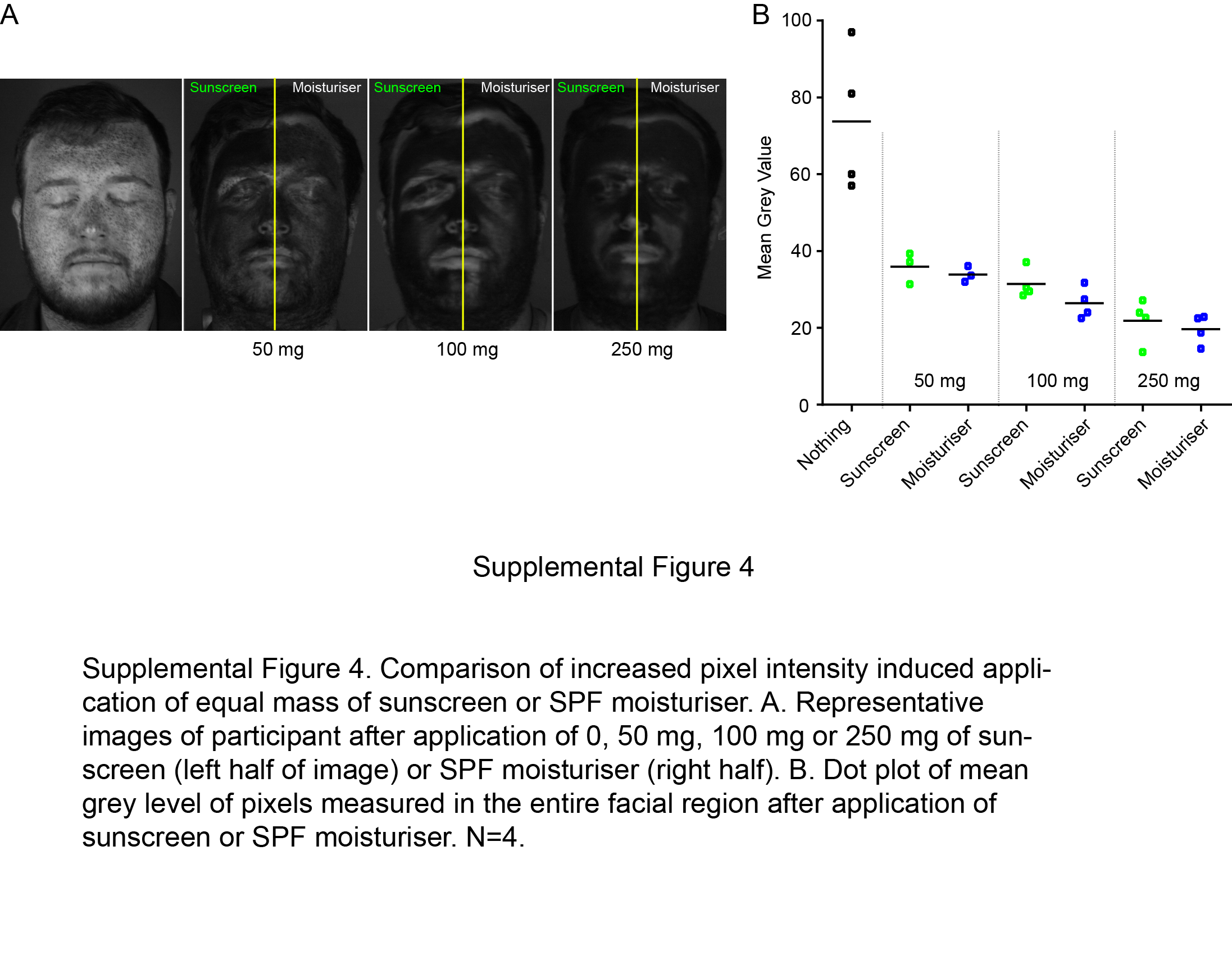

Supplement: S4 Fig — A. Representative images of participant after application of 0, 50 mg, 100 mg or 250 mg of sunscreen (left half of image) or SPF moisturiser (right half). B. Dot plot of mean grey level of pixels measured in the entire facial region after application of sunscreen or SPF moisturiser. N = 4. (TIF) [file pone.0212548.s004.tif]

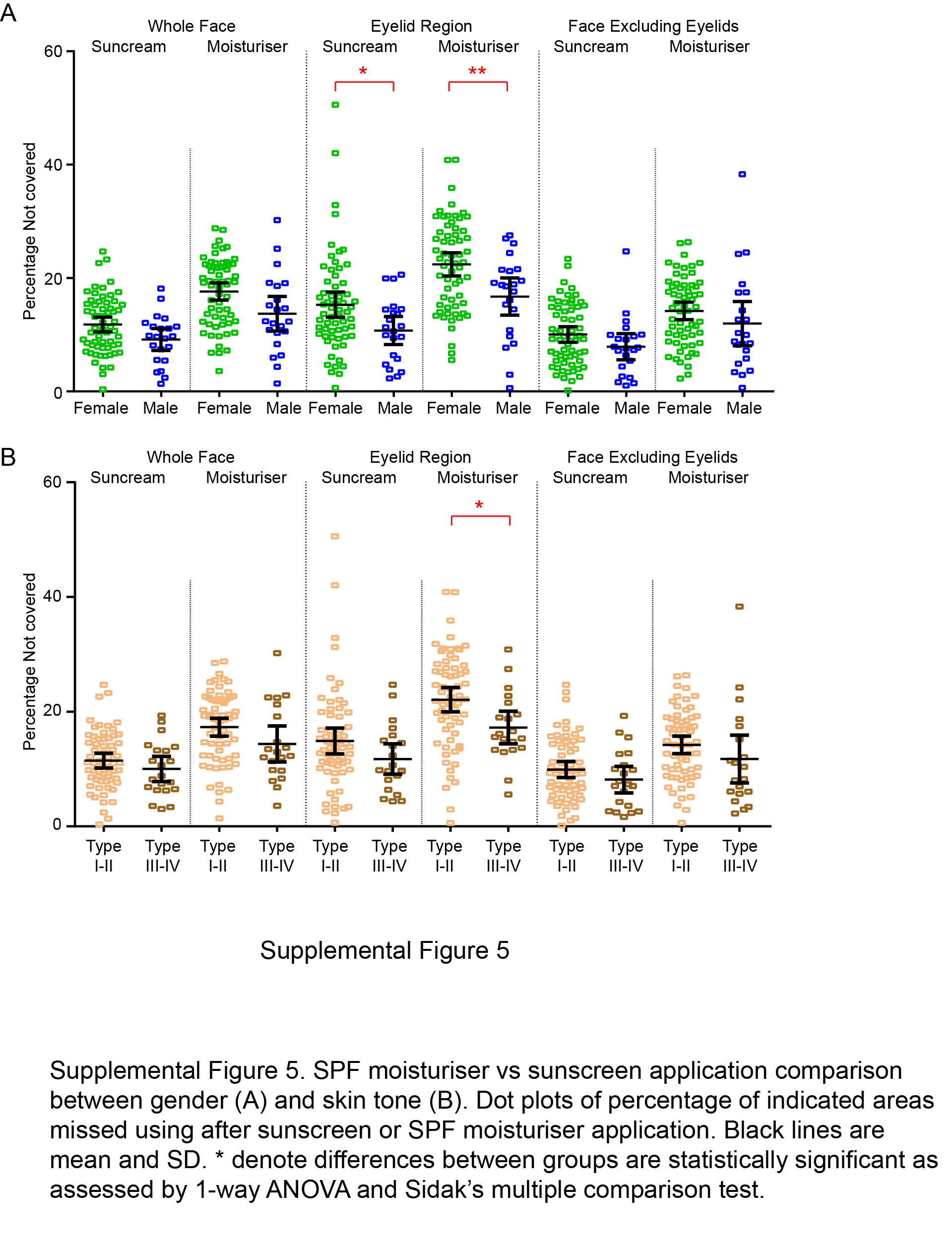

Supplement: S5 Fig — Dot plots of percentage of indicated areas missed using after sunscreen or SPF moisturiser application. Black lines are mean and standard deviation. * denote differences between groups are statistically significant as assessed by 1-way ANOVA and Sidak’s multiple comparison test. (TIF) [file pone.0212548.s005.tif]

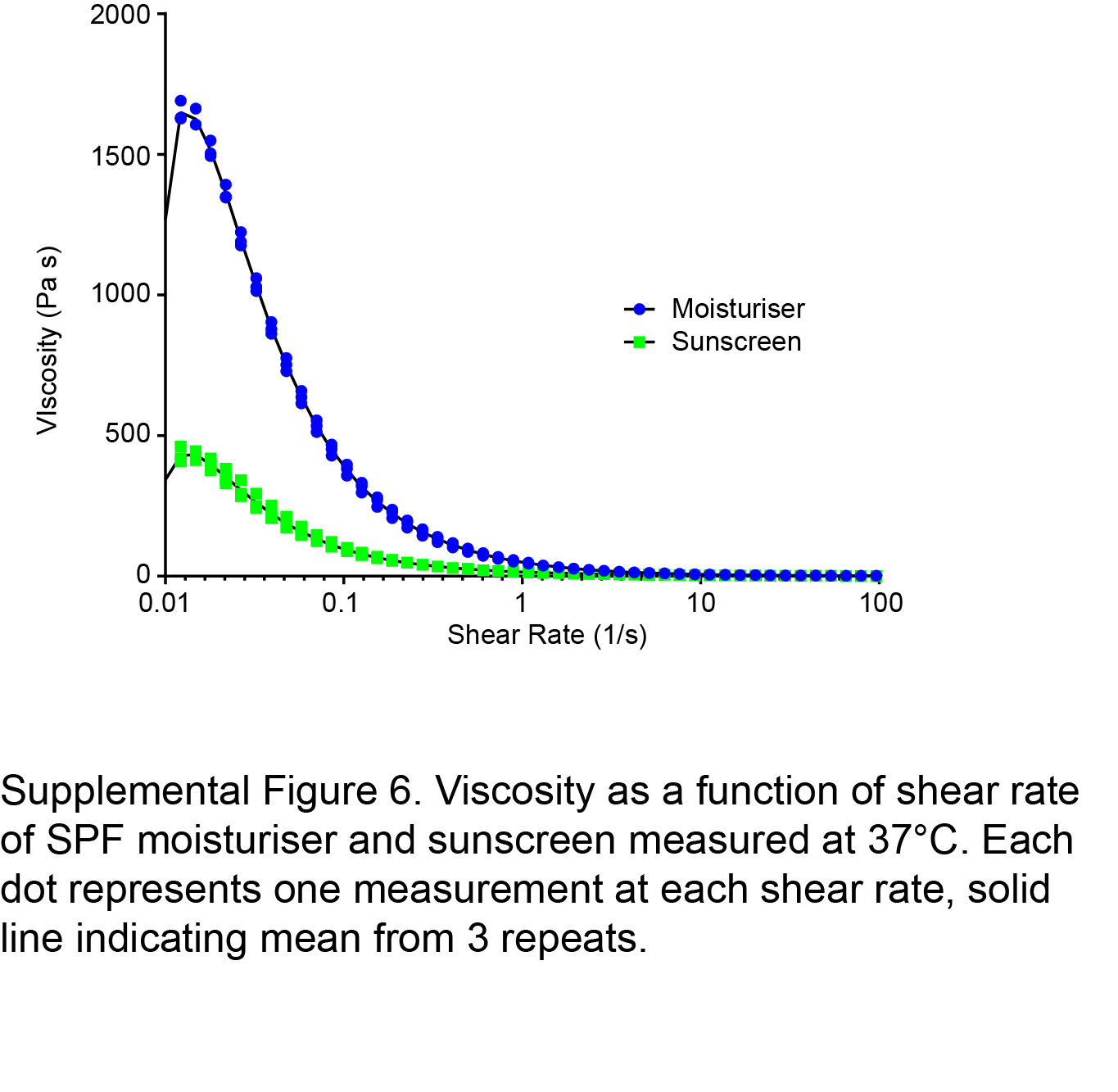

Supplement: S6 Fig — Each dot represents one measurement at each shear rate, solid line indicating mean from three repeats. (TIF) [file pone.0212548.s006.tif]
